# A dual function TAR Decoy serves as an anti-HIV siRNA delivery vehicle

**DOI:** 10.1186/1743-422X-7-33

**Published:** 2010-02-10

**Authors:** Hoshang J Unwalla, John J Rossi

**Affiliations:** 1 Department of Microbiology and Immunology, Miller School of Medicine, University of Miami, Miami Fl 33136, USA; 2 Division of Molecular Biology, Beckman Research Institute of the City of Hope, Graduate School of Biological Sciences, Duarte CA 91010, USA

## Abstract

The TAR RNA of HIV was engineered as an siRNA delivery vehicle to develop a combinatorial therapeutic approach. The TAR backbone was found to be a versatile backbone for expressing siRNAs. Upon expression in human cells, pronounced and specific inhibition of reporter gene expression was observed with TARmiR. The resulting TARmiR construct retained its ability to bind Tat and mediate RNAi. TARmiR was able to inhibit HIV gene expression as a TAR decoy and by RNA interference when challenged with infectious proviral DNA. The implications of this dual function therapeutic would be discussed.

## Background

Since its discovery in the eighties, significant progress has been made in attempts to control HIV. Several strategies have been adopted including the use of small molecule drugs to inhibit various stages in the viral life cycle collectively called a HAART regimen. Unfortunately, nearly all HIV-infected individuals on HAART will need to maintain their medications for the entirety of their lives, resulting in considerable expense and sometimes the toxic side effects of these drugs. According to recent reports, in the age of HAART the majority of emergency room visits by HIV-infected individuals has shifted from opportunistic infections to treatments for antiretroviral drug-related toxicities. Thus there is a clear need to develop alternative therapies to treating HIV infections. Alternative approaches to inhibit HIV have explored the use of a genetic type of therapy where HIV susceptible T-cells or stem cells that are precursors of HIV susceptible cells have been engineered to express anti-HIV molecules. These include oligonucleotide-based antivirals like siRNA, ribozymes, suicide genes or transdominant negative mutant proteins of HIV. Many of these approaches have shown promise at restricting viral replication. Some genetic therapy approaches have also progressed to clinical trials. However a serious limitation with designing anti-HIV therapies is the ability of the virus to evolve and become resistant to any one therapeutic approach. This is due to the low fidelity of the HIV reverse transcriptase. Hence it is essential to develop intervention strategies that can significantly restrict the ability of the virus to become resistant to it. One way this can be achieved is by using an approach where two or more inhibitors are used in combination such that if the virus manages to become resistant to one, it is inhibited by another. Another escape proof strategy is to interfere with normal viral RNA protein interactions that are critical in HIV life cycle namely the Tat-TAR interaction or the Rev RRE interaction. Indeed several studies including ours have explored the use of TAR or RRE decoys [1-3] or the use of transdominant negative mutants of rev[4,5]. We have earlier reported a combinatorial approach where an anti-HIV siRNA is co-expressed along with Rev M10, a transdominant negative mutant of HIV rev to effect a pronounced inhibition of HIV, concomitantly suppressing the emergence of viral mutants in T-cell lines[6].

RNAi mediated gene silencing can be achieved by either transfecting dsRNA [7,8] or plasmids expressing the siRNA either as sense and antisense strand or as a hairpin[9,10]. The proteins involved in RNAi are evolutionarily conserved and play a role in silencing of developmentally important genes. siRNAs exploit the an endogenous miRNA pathway to mediate RNAi. MicroRNAs (miRNAs) are an important class of small, noncoding, regulatory RNAs found to be involved in regulating a wide variety of important cellular processes by the sequence-specific inhibition of gene expression. They serve important regulatory functions in a variety of cellular processes, including differentiation, development, and metabolism (For review see [11-13].

Some studies have also reported that siRNAs expressed from a microRNA backbone could efficiently inhibit cognate gene expression[14]. Cloning of small RNAs from viruses demonstrated the presence of microRNAs encoded by viruses [15-17]. miRNAs have several characteristics that make them an attractive option for viruses to utilize in the regulation of gene expression. miRNAs could be envisioned to function in viral pathogenesis in several ways, including the regulation of viral gene expression by host miRNAs, the regulation of viral gene expression by virus encoded miRNAs, and the regulation of host genes by virus encoded miRNAs. Recently Ouellette et. al. [18] reported the processing and release of functional microRNAs from the HIV transactivation response element (TAR). They further went on to report that the processed microRNA can mediate RNA interference.

TAR element is a structured RNA located at the 5' end of all transcripts derived from HIV-1[19,20]. It is a master switch that turns ON HIV replication. By interfering with the Tat-TAR interaction one can have an amplifying effect whereby the viral transcription never takes off. Michienzi et. al. have reported a robust inhibition of viral replication by expressing a nucleolar localized TAR decoy[3].

In this study we report the expression of an anti-HIV siRNA from the TAR RNA backbone. We further go on to demonstrate that the anti-HIV Tar-miRNA construct can function as dual-function therapeutic serving as a TAR decoy as well as an siRNA delivery vehicle. This dual function anti-HIV TARmiR causes potent inhibition of HIV gene expression when delivered directly or expressed in HIV infected cells. The potential advantages and applications of this system would be discussed.

## Results

### Anti-HIV TARmiR inhibits cognate gene expression

**Figure 1:**
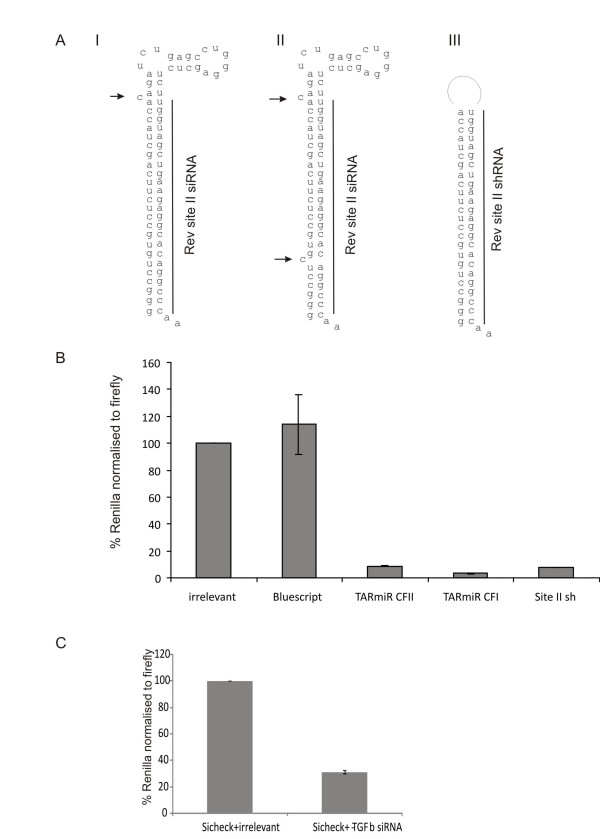
**Inhibition of target gene expression by siRNAs expressed from the TAR microRNA backbone**. (A) siRNA sequence targeting the earlier reported site II of HIV rev was folded in silico and the configurations that retained the correct Tat binding region were selected for further studies. Configurations I & II have the lower single nucleotide bulge removed or both the bulges retained respectively. Configuration III is the anti-site II Rev shRNA with the 9 nt loop reported earlier by us[28]. Arrows indicate single nucleotide bulge (B) Target site corresponding to the site II of HIV rev is cloned in the 3' untranslated region of the renilla luciferase ORF in the siCHECK vector. The three configurations of siRNA were invitro transcribed and treated with Calf intestinal alkaline phosphatase. The CIP treated RNA were then cotransfected with the psiCHECK plasmid having the Rev Target site. Dramatic inhibition of reporter gene expression is observed with all three configurations. The configuration where the lower bulge is removed is three-fold more potent than even the anti-Rev shRNA. (C) An siRNA targeting TGF-β is expressed in a similar fashion from the TAR miRNA backbone and co-transfected with siCHECK plasmid having the TGF-β target site. ~70% inhibition is observed with this construct suggesting that the TAR miRNA is a versatile backbone for expressing siRNA. All siCHECK assays are a mean of three experiments. CFI and CFII = Configuration I and Configuration II.

To determine if an anti-HIV siRNA can be expressed in the context of the HIV TAR element, siRNA sequence corresponding to the earlier reported site II of HIV Rev was inserted in place of the cognate TAR miRNA sequence. It is also essential to retain the proper folding of the TAR bulge to ensure that the TARmiR can also function as a TAR decoy. Several configurations of TAR were designed and folded in silico to determine if the TAR stem folds correctly. In one configuration, a perfect stem corresponding to the sequence of the siRNA was incorporated in TARmiR. The modification alters the TAT binding region of TAR and has been reported to inhibit TAT binding (TARmiR- perfect stem) [21]. In order to retain the correct folding of TAR, two configurations were selected, one in which both the single-nucleotide bulges are retained as in the wild type TAR and another in which the distal bulge near the Tat binding region is retained (Figure 1A). The anti- rev siRNA target site corresponding to our previously reported site II was cloned in the siCHECK plasmid (Promega). The TAR microRNAs were in vitro transcribed using the T7 transcription kit from Promega. Earlier reports have indicated that T7 transcribed RNA can activate a non-specific innate immune response [22]. To prevent this and to ensure that inhibition of HIV gene expression is due to the siRNA effect, the TAR miRNA was treated with Calf intestinal alkaline phosphatase (CIAP) to remove the initiating triphosphate. The CIP treated anti-site II rev TARmiR were then co-transfected with the siCHECK plasmid carrying the rev target in the 3' UTR of Renilla luciferase. 48 hours post-transfection the cells were harvested and the luciferase activity measured according to the manufacturer's instructions. siCHECK transfected with a similarly transcribed anti-site II rev shRNA was used for comparison. As seen in figure 1B, ~80% inhibition of the target is observed with the in vitro transcribed shRNA. A comparable inhibition is observed with the configuration where both the single-nucleotide bulges are retained. The configuration where the proximal bulge is removed showed a dramatic inhibition three fold better than the in vitro transcribed shRNA suggesting that the siRNA expressed from this configuration was much more potent than expressing it from a hairpin. This could be expected since TAR is naturally processed into microRNA and hence is a natural substrate for DICER. TAR is also known to recruit the TAR RNA binding protein (TRBP), which is an important component of the RNAi machinery. In order to determine if the presence of Tat binding region of TAR contributes to RNAi in any way, a perfect stem with the TAR sequence minus the bulges, TARmiR-perfect stem, was separately tested as a control. The TARmiR-perfect stem construct inhibited target gene expression in siCheck Assays with an efficiency comparable to that observed with anti-Rev shRNA (Additional file 1) To determine the versatility of the TAR RNA backbone for expressing siRNA, we replaced the Rev site II with a site for TGF-β gene. This anti-TGF-β TARmiR was similarly transcribed and co-transfected with siCheck plasmid carrying the TGF-β target site. As seen in figure 1C, ~60% inhibition of target gene expression is observed. The inhibition by both, the anti-site II rev and anti-TGF-β TARmiR RNA was specific to its cognate siCHECK targets. No inhibition was observed by anti-TGF-β TARmiR for the HIV rev target (Additional file 2; 2A). Neither of the two anti-HIV TARmiR configurations nor the anti-rev shRNA demonstrated any inhibition of siCHECK plasmid having a TGF-b site cloned as target (Additional file 2; 2B). Thus the TAR backbone serves as a versatile expression vehicle for siRNA.

### Anti-HIV TARmiR functions via the siRNA pathway

**Figure 2:**
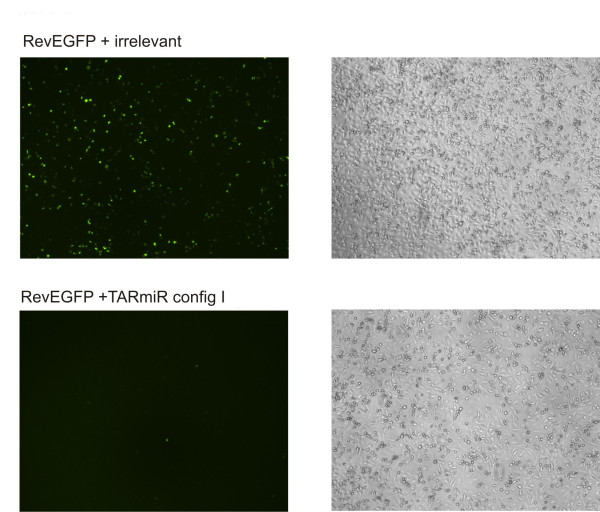
**anti- rev TARmiR configuration I can function as siRNA in RNA interference: HEK 293 cells were co-transfected with CMV RevEGFP and the anti-HIV TARmiR configuration I**. Potent inhibition of EGFP expression is observed in these cells suggesting that the siRNA sized fragments released from the TARmiR backbone can demonstrate RNAi even when the target site is present within the ORF.

Since the siCheck system involves cloning the target in the 3' UTR of the Renilla luciferase, the system is ideal for detecting both an siRNA and an miRNA effect. However it is quite possible that the siRNA sized fragment released from TARmiR could have a potent miRNA effect that is readily detectable by siCHECK but may not work if the target is within the open reading frame (as an siRNA). To test whether the TARmiR can inhibit HIV rev as an siRNA, CMV RevEGFP plasmid that encodes a full-length functional Rev fused to the EGFP sequence was co-transfected with the distal single-nucleotide bulge containing configuration of TARmiR. As seen in figure 2, a potent inhibition of RevEGFP expression is observed in presence of the TARmiR. Cells receiving irrelevant siRNA did not show any inhibition.

### Anti-site II rev TARmiR can bind Tat

**Figure 3:**
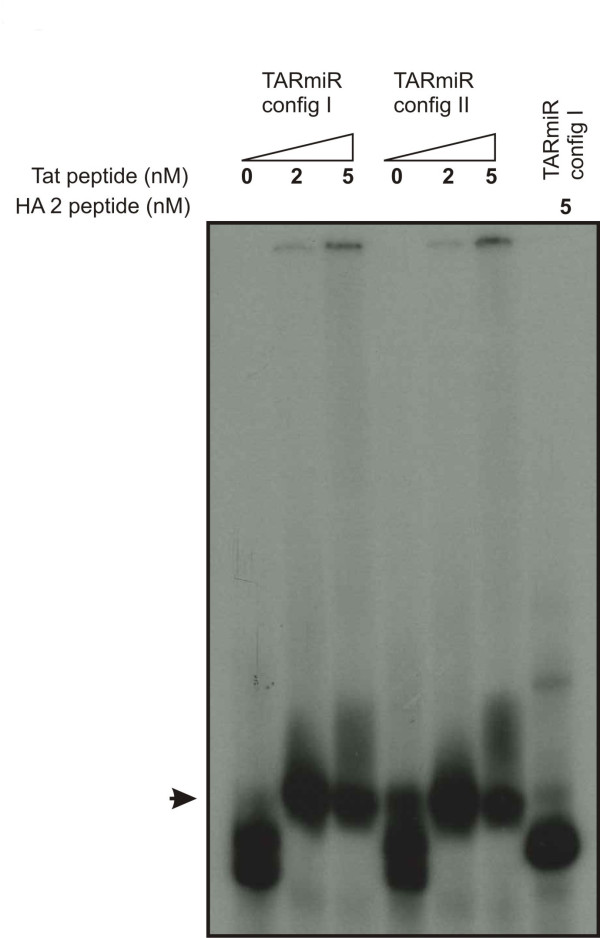
**Gel mobility shift assay: anti-HIV TARmiR configuration I or II was transcribed in vitro, end labeled and was allowed to bind to a peptide corresponding to the arginine rich region of Tat that is responsible for binding TAR **[23]. A mobility shift (arrow) clearly demonstrates Tat peptide binding to the TARmiR. As a control the fusion peptide HA-2 of influenza was used to bind the TARmiR configuration I. No shift in mobility is observed with HA2.

To determine if either of the TARmiR configurations bind Tat and can potentially serve in the dual role of a TAR decoy and an siRNA vehicle, both the configurations were end-labeled and allowed to bind a previously reported Tat derived peptide corresponding to the region of Tat that binds TAR [23]. To determine if the binding is TAT specific, the influenza HA2 fusion peptide was allowed to bind TARmiR configuration I. As seen in figure 3, a mobility shift is observed with both the configurations while the HA-2 peptide does not show any binding. This validates our in silico folding data demonstrating that both the configurations fold correctly and retain their TAT binding abilities and can potentially function as a TAR decoy as well as function in RNAi.

### Inhibition of HIV gene expression by anti-HIV TARmiR expressed from a U6 promoter

**Figure 4:**
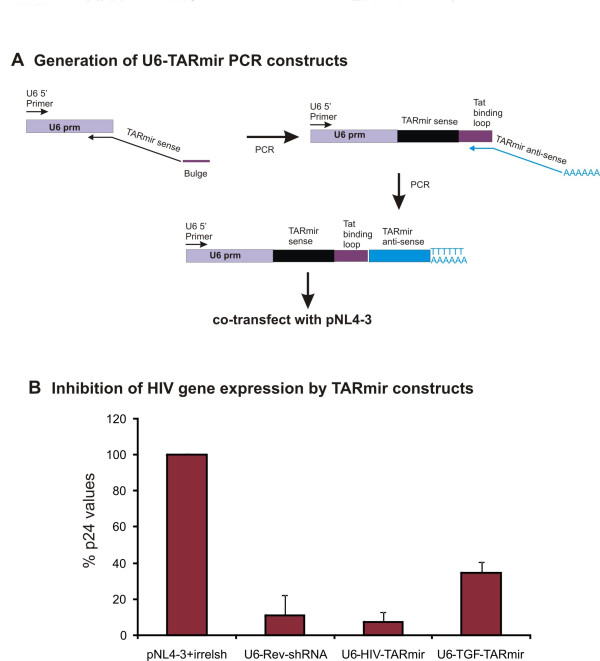
**Inhibition of HIV gene expression by TARmiR constructs**. (A) Generation of U6-TARmiR PCR constructs. An PCR expression cassette with the U6 promoter driving the expression of anti-rev TARmiR, anti-TGF-β TARmiR or anti-Rev shRNA is generated as described earlier by us [24]. (B) HEK 293 cells are co-transfected with infectious proviral DNA pNL4-3 and U6anti-Rev TARmiR configuration I, U6 anti-TGF-β TARmiR or U6anti-Rev shRNA. Pronounced inhibition is observed with both, the anti-Rev shRNA and anti-Rev TARmiR. Inhibition is also observed with anti-TGF-β TARmiR. This could be due to the presence of an intact TAT binding bulge which serves in the capacity of a TAR decoy.

To determine if the TARmiR construct can serve as a dual function therapeutic when expressed in cells, we used a PCR based approach for rapid synthesis of U6 promoter-TARmiR constructs as reported earlier by our laboratory [24]. Both the Rev site II containing and TGF-β containing TARmiR were similarly generated (Fig 4A). HEK 293 cells were co-transfected with the infectious proviral DNA, pNL4-3 and U6 promoter PCR cassettes containing either irrelevant shRNA, anti-Rev containing TARmiR, anti-TGF-β containing TARmiR or Rev site II shRNA. Culture supernatants were collected on day3 and the p24 levels in the supernatant were determined using a p24 ELISA kit. As seen in figure 4B, inhibition is observed with all the U6 constructs with maximal inhibition observed with the anti-HIV shRNA and anti-HIV TARmiR. Some inhibition is also observed with the anti-TGF-β TARmiR. This inhibition could be attributed to the presence of the TAR bulge that would serve as a TAR decoy and hence sequester Tat thereby down-regulating transcription from the LTR of pNL4-3. This demonstrates that expressing anti-HIV siRNA from the TAR backbone can have a dual impact on HIV replication in that the TAR bulge could serve as a TAR decoy whereas the processed siRNA can inhibit HIV via RNA interference.

## Discussion

Here we show that siRNA expressed from HIV TAR backbone successfully inhibits HIV by mediating RNAi as well as serving as a TAR decoy. Several configurations of TARmiR were designed and folded in-silico to determine if placing the anti-HIV rev siRNA within the TAR backbone alters the correct TAR folding. It is essential to preserve the correct structure of the TAR bulge to facilitate TAT binding for the TARmiR to serve as a TAR decoy. It was determined that replacing both the single nucleotide bulges in the TAR stem loop with a perfect stem alters the structure of the TAR bulge (data not shown). Two configurations of TAR were tested which both retained the structure of the TAR loop, one in which both bulges were retained as in the original HIV TAR and the other where only the distal bulge is retained. Both these configurations demonstrated pronounced inhibition of reporter gene expression. The distal single nucleotide bulge-containing configuration was three times more potent than the configuration with both the bulges as well as a conventional shRNA targeting the same site. We were able to demonstrate target knockdown both, when the target is in the 3' UTR of the reporter gene as well as when the target is within the ORF as seen with inhibition of RevEGFP expression. Both the configurations retained their ability to bind HIV tat as demonstrated by gel mobility shift analysis. The binding was specific since an unrelated peptide of influenza virus failed to bind this configuration. The engineered TAR retained its ability to bind HIV tat and demonstrated a TAR decoy effect when an unrelated siRNA was expressed from its backbone in co-transfection experiments with infectious HIV proviral DNA.

Several reports including one from our laboratory have demonstrated efficient inhibition of HIV gene expression using TAR decoys[3]. The Tat-TAR interaction is very critical for HIV and serves as a master switch that turns ON gene expression. While the HIV LTR is very efficient at transcription initiation, RNA polymerase II is non-processive. It transcribes TAR and pauses at the base of the TAR loop. HIV TAT binds TAR and recruits the transcription factor PTEF-b kinase, which is a heterodimer of CDK-9 and cyclin T1. CDK9 phosphorylates the C-terminal domain of Pol II and makes it processive allowing the transcription to proceed. However binding of NF-kb subunits in response to cellular events or signal transduction can also result in efficient initiation and elongation of transcription. Of note the p65 subunit of NF-kb can make the Pol II elongation competent. Thus allowing a lower level of transcription to proceed even in absence of Tat.

Expressing an siRNA from the backbone of TAR can provide the second tier of inhibition and target transcription that is TAT independent or, in case the TAR decoy is overwhelmed by excessive transcription from the HIV LTR. Moreover such a construct would provide a single RNA molecule that can target HIV in two different ways. The ability to do so can simplify issues with expression of these as a transgene as in case of gene therapy or delivery of this RNA molecule to HIV infected/susceptible cells when coupled to either T-cell specific monoclonal antibodies[25] or HIV gp160 aptamers [26]. Indeed when combined with anti-HIV aptamers as demonstrated by Zhou et. al. [26] one can create a single RNA molecule that inhibits HIV in three distinct ways where the gp120 aptamer can neutralize the free virus or bind to infected cell surface and block cell-cell fusion, the TAR bulge can serve as a TAR decoy and the siRNA can target the HIV transcript. In our earlier work we have demonstrated that targeting HIV with shRNA alone can allow selection of mutants that are resistant to the shRNA[6]. Such mutants are observed within 40 days of culturing the virus with cells stably expressing these shRNA. However when a combinatorial approach was used where the siRNA was co-expressed with the transdominant negative mutant of rev (RevM10) we observed an additive effect and suppressed the emergence of resistant mutants. It is quite possible that the TARmiR can serve primarily as a TAR decoy and binding of tat to TARmiR can block processing by DICER, meaning that a molecule of TARmiR can either serve as a TAR decoy or get processed to functional siRNA but not both, we do not anticipate that to be a limitation of this design since sufficient molecules of TARmiR would be made available either by expression or delivery such that while some molecules would bind Tat and serve as a TAR decoy others would still be available to get processed and mediate RNAi. Future work would revolve around replacing the shRNA in our earlier reported co-expression cassette with anti-site II Rev TARmiR and co-expressed with revM10 to deliver a triple blow to HIV from a single transgene cassette. We anticipate a pronounced inhibition of HIV gene expression using this cassette, which would also be HIV inducible. Alternately these anti-HIV TARmiR coupled to gp120 aptamers for delivering them directly to HIV infected cells.

## Materials and methods

### Materials

Unless otherwise noted, all chemicals were purchased from Sigma-Aldrich, all restriction enzymes were obtained from New England Biolabs (NEB) and all cell culture reagents were purchased from GIBCO (Invitrogen). The Tat 48-57 peptide with the sequence YGRKKRRQRRRP and HA-2 fusion peptide GLFEAIAGFIENGWEGMIDGK were purchased from American peptide Company (Sunnyvale CA).

### Plasmids

Infectious proviral DNA clone pNL4-3 was obtained from the NIH AIDS reagent and Reference program, Division of AIDS, NIAID, NIH. psiCHECK-2 Plasmid was obtained from Promega corporation. To generate siCHECK Plasmids having the rev site and TGF-β site, DNA sequence corresponding to the siRNA sense strand and its antisense strand with an Xho I site and Not I site overhang was synthesized chemically, annealed, digested with Xho I and Not I and ligated into a similarly digested psiCHECK-2 plasmid.

### Cell Culture

HEK 293 cells were purchased from American Type Culture Collection and cultured in Dulbecco's modified eagle's medium supplemented with 10% fetal bovine serum in accordance with its respective data sheet. All transfections were done using Lipofectamine 2000 reagent (Invitrogen) according to the manufacturer's instructions.

### Generation of TARmiR constructs

All DNA oligonucleotides were purchased from Sigma. The T7-TARmiR expression cassettes were generated as described below.

Anti-Rev site II TARmiR configuration I:

Sense primer: *Taatacgactcactata *gggcctgtgcctcttcagctaccacagatctgagcctggga

Antisense Primer: ttgggcctgtgcctcttcagctaccaagagagctcccaggctcagatctgtg

Anti-rev site II TARmiR configuration II:

Sense primer: *Taatacgactcactata *gggcctcgtgcctcttcagctaccacagatctgagcctggga

Antisense primer: ttgggcctgtgcctcttcagctaccaagagagctcccaggctcagatctgtg

Anti-TGF-β TARmiR configuration I

Sense primer: *Taatacgactcactata *gggcatgtcatcagctgggaagacagatctgagccctggga

Antisense primer: ttgggcatgtcatcagctgggaagaagagagctcccagggctcagatctgtcttc

TAR-miR-perfect stem configuration:

Sense primer: *Taatacgactcactata *gggcctgtgcctcttcagctaccttcatctgagcctggga

Antisense primer: ttgggcctgtgcctcttcagctaccaagagagctcccaggctcagatg

The sense and antisense primers were annealed as described

Annealing Mix: 9 μl 100 mM Tris-HCl, pH 8.0 15 μl 50 mM MgCl2, 1 nmole sense primer, 1 nmole antisense Oligo, total volume of 90 μl with MQ H2O

In a separate tube, 50 μl 10× PCR Buffer (without Mg), 4 μl 25 mM each dNTP, 2 μl Platinum Taq, 354 μl MQ H2O, Divide into 5 × 82 μl reactions. All tubes were heated to 93°C and then allowed to cool to room temperature. Distribute 18 μl of the annealing reaction into each of the Platinum Taq mixtures.

Extension reaction is carried out at 72°C for 10 minutes. The extension products are purified using Qiagen PCR purification columns and the size confirmed by running on agarose gel electrophoresis.

### In vitro transcription

The primer extension products are then used for in vitro transcription using the RiboMAX Large Scale RNA Production System-T7 (Promega). For end-labeling reaction, TARmiR RNA was 5' labeled with [γ-32P]ATP (7000 Ci/mmol; MP Biomedicals) and T4 polynucleotide kinase as previously described [27]

### Dual luciferase assays

HEK 293 cells were transfected with 100 ngs of siCHECK plasmid containing either the rev site II or TGF-β target site and 10 pmoles of in vitro transcribed either anti-site II rev or TGF-β TARmiR. 48 hours post-transfection, cells were harvested for analysis. The expression of Renilla luciferase and normalizing control Firefly luciferase were detected using the Dual-luciferase reporter assay system (Promega, Madison, WI), in accordance with the manufacturer's instructions. All samples were transfected in triplicate, and the experiment was performed a minimum of three times.

### Gel retardation assay

TARmiR Configuration I and II were invitro transcribed and labeled as mentioned above. For binding reaction Peptide (TAT or HA2) and RNA were incubated together for 10 min on ice in 10-ml binding reactions containing 10 mM Tris-HCl (pH 7.5), 70 mM NaCl, 0.2 mM EDTA, and 5% glycerol. Peptide-RNA complexes were resolved on 10% polyacrylamide, 0.5 × TBE gels that had been prerun for 1 hr. Gels were electrophoresed at 200 V for 3 hr at 4°C, dried, and exposed to an X-ray Film.

### HIV challenges and p24 antigen assay

HEK 293 cells were co-transfected with the infectious proviral DNA clone, pNL4-3 and the anti-site II rev TARmiR, anti-TGF-β TARmiR or anti-site II rev shRNA. 72 hours post-transfection, culture supernatants were collected. The p24 antigen analyses were performed using a Coulter HIV-1 p24 antigen assay (Beckman Coulter, Fullerton, CA) in accordance with the manufacturer's instructions.

## Competing interests

The authors declare that they have no competing interests.

## Authors' contributions

HU is the corresponding author. JR is the co-corresponding author. HU and JR conceived of the study. HU did all the experiments and drafted the manuscript. All authors read and approved the final manuscript.

## Supplementary Material

Additional file 1 **Inhibition of Target RNA expression with the minus bulge control shows ~85% inhibition.** The inhibition is comparable to that observed with anti-Rev shRNA. Click here for file

Additional file 2 **HEK 293 cells were co-transfected with either of the anti-HIV TARmiR configurations or anti-HIV shRNA and siCheck plasmid having the TGF-β target site (A) or vice versa (B).** Dual Luciferase assay was performed as described in Materials and Methods. As seen in the figure none of the TARmiR configurations or anti-Rev shRNA demonstrated any inhibition of the non-cognate siCHECK. Click here for file
