# Supplementary figures and images for: A dual function TAR Decoy serves as an anti-HIV siRNA delivery vehicle
Source: Virol J. 2010 Feb 10;7:33. doi: 10.1186/1743-422X-7-33 (PMC2836314; doi:10.1186/1743-422X-7-33)

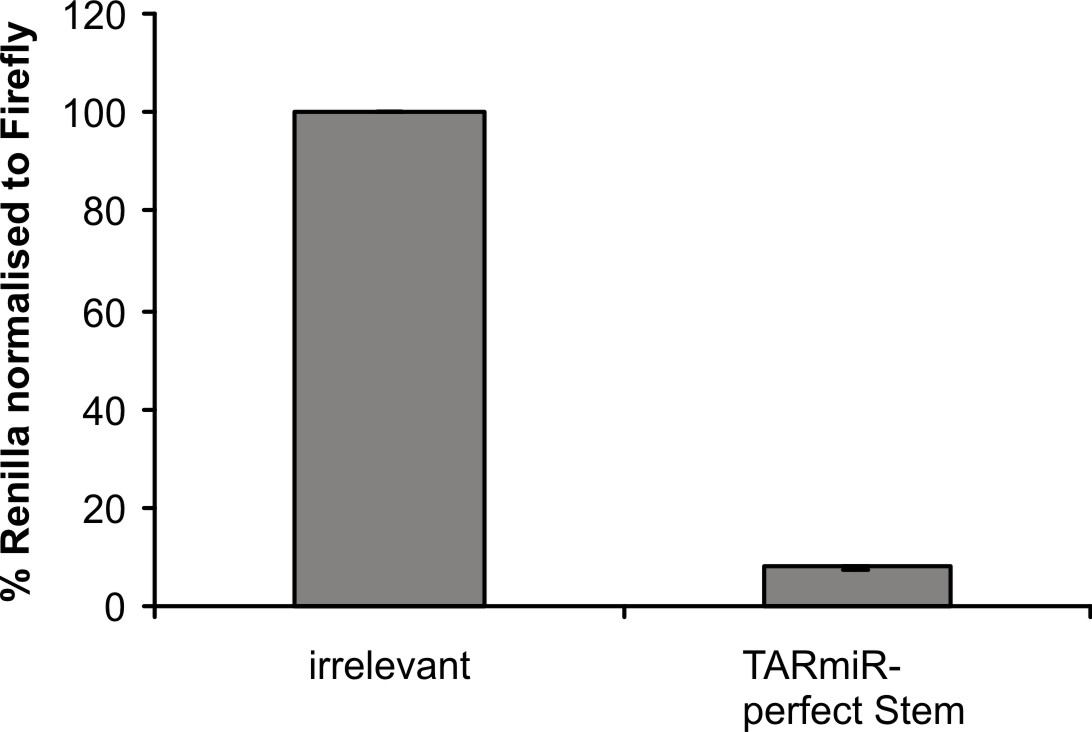

Supplement: Additional file 1 — Inhibition of Target RNA expression with the minus bulge control shows ~85% inhibition. The inhibition is comparable to that observed with anti-Rev shRNA. [file 1743-422X-7-33-S1.JPEG]

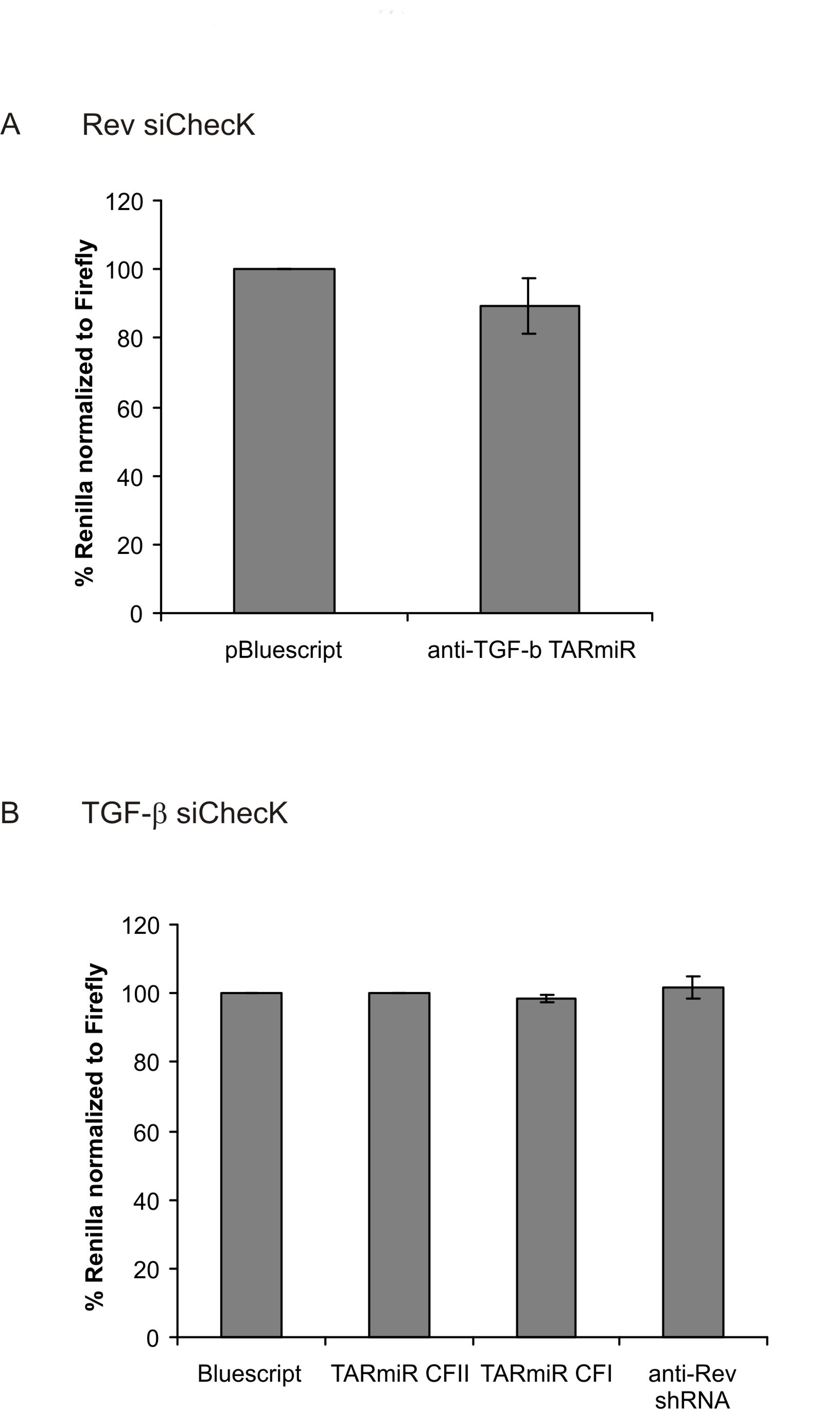

Supplement: Additional file 2 — HEK 293 cells were co-transfected with either of the anti-HIV TARmiR configurations or anti-HIV shRNA and siCheck plasmid having the TGF-β target site (A) or vice versa (B). Dual Luciferase assay was performed as described in Materials and Methods. As seen in the figure none of the TARmiR configurations or anti-Rev shRNA demonstrated any inhibition of the non-cognate siCHECK. [file 1743-422X-7-33-S2.JPEG]
